# Cyclic RGD Peptide Targeting Coated Nano Drug Co-Delivery System for Therapeutic Use in Age-Related Macular Degeneration Disease

**DOI:** 10.3390/molecules25214897

**Published:** 2020-10-23

**Authors:** Jiaxin Liu, Lifu Luo, Fei Xu, Ge Li, Jicong Chen, Lesheng Teng, Youxin Li, Fengying Sun

**Affiliations:** 1 School of Life Sciences, Jilin University, Changchun 130012, China; jxliu328@163.com (J.L.); xufei19@mails.jlu.edu.cn (F.X.); lige18@mails.jlu.edu.cn (G.L.); chenjc20@mails.jlu.edu.cn (J.C.); tenglesheng@jlu.edu.cn (L.T.); 2 Department of Ophthalmology, Second Clinical College of Norman Bethune Medical Division, Jilin University, Changchun 130041, China; luolf@jlu.edu.cn

**Keywords:** vascular endothelial growth factor, age-related macular degeneration, anti-angiogenic drug, retinal pigment epithelial cells, nanoparticles

## Abstract

Vascular endothelial growth factor (VEGF) expression increased significantly in the pathogenesis of age-related macular degeneration, which induced the formation of pathological blood vessels. Dexamethasone is an exogenous anti-angiogenic drug while bevacizumab is an endogenous anti-angiogenic drug. They both have been widely used in ophthalmology. However, independent administration is not enough to completely block the development of choroidal neovascularization (CNV), and the number of eyes vitreous injections is limited. Reasonable combination of drugs may produce significantly better therapeutic effect than single drug treatment. The cyclic RGD (cRGD) peptide has a particularly high affinity with retinal pigment epithelial cells, where VEGF secretes from. In this study, we prepared nanoparticles of bevacizumab and dexamethasone with cRGD peptide as the target (aBev/cRGD-DPPNs). The particle size of the aBev/cRGD-DPPNs was 213.8 ± 1.5 nm, SEM results showed that the nano-carriers were well dispersed and spherical. The cell uptake study demonstrated the selectivity of the aBev/cRGD-DPPN to ARPE-19 with α_V_β_3_ over expressed. The aBev/cRGD-DPPNs had a better apoptosis induction effect and an obvious inhibitory effect on migration, invasion, and capillary-like structures formation of human umbilical vein epithelial cells. The fluorescein fundus angiography study, immunohistochemistry and histopathological evaluation showed the aBev/cRGD-DPPNs greatly reduced the development of CNV on a rabbit model.

## 1. Introduction

According to the World Health Organization, there are about 1.3 billion people suffering from a certain degree of visual impairment and 36 million are blind worldwide [1]. Retinal diseases have become the main cause of blindness, among which age-related macular degeneration (AMD) is the leading cause. AMD is mainly divided into dry AMD and wet AMD [2,3]. Dry AMD is also known as atrophic AMD, which is clinically manifested as progressive pigment epithelial atrophy. Wet AMD is also called neovascular AMD, which is clinically manifested as active neovascularization under pigment epithelial layer. Although patients with dry AMD are much more than those with wet AMD, wet AMD has a high risk of blindness in a short period of time and more than 90% of patients with acute visual impairment, so wet AMD is paid more attention [4]. Choroidal neovascularization (CNV) is the main cause of visual impairment in patients with wet AMD [5].

At present, the most common drug treatment for CNV is anti-angiogenic drugs, which are mainly divided into endogenous anti-angiogenic drugs and exogenous anti-angiogenic drugs [6,7]. Glucocorticoids, as exogenous antiangiogenic drugs, are commonly used in clinical ophthalmology [8,9,10]. Dexamethasone is the most commonly used glucocorticoid and is widely used in the treatment of various diseases [11]. Studies have shown that dexamethasone can promote the regression of choroidal neovascularization by releasing local noradrenaline to constrict blood vessels, reducing or silencing the expression of vascular endothelial growth factor (VEGF), inducing the apoptosis of vascular endothelial cells and inhibiting the proliferation of vascular endothelial cells [12]. Vitreous administration is required due to its systemic side effects and the unique barrier structure of the eyeball [13,14]. However, in view of the fast metabolism of dexamethasone, the vitreous administration is not suitable for frequent operation due to pain and side effects [15,16,17]. Therefore, the development of suitable carriers will reduce the drug injection frequency and improve the selectivity of dexamethasone.

Endogenous antiangiogenic drugs mainly include various cytokines and cytokine antibodies or antagonists and receptor blockers [18], VEGF is the major cytokine for CNV development [19,20]. Clinically, existing anti-VEGF drugs have achieved a certain effect in the treatment of neovascular eye diseases [21,22]. Bevacizumab is the first approved anti-tumor angiogenesis drug in the USA for the treatment of metastatic colorectal cancer [23]. In recent years, bevacizumab has been widely used as “off-label” by ophthalmologists because of its efficacy in treating underlying ophthalmic diseases as well as its low cost and efficacy in the treatment of fundus diseases, which has been proved by clinical practice [24]. However, due to the complex pathogenesis of AMD, bevacizumab is not enough to completely block the development of CNV, and the number of vitreous injections that the human eye can bear is limited. Reasonable combination of drugs may produce significantly better therapeutic effect than single drug treatment.

The pathological mechanism of CNV is the dysfunction of retinal pigment epithelial cells (RPE), which releases excessive VEGF into the choroid, and excessive VEGF can induce the formation of pathological new blood vessels of AMD [25,26]. The secretion of VEGF mainly comes from retinal pigment epithelial cells (RPE), so RPE are potential targets for reducing VEGF expression and preventing CNV formation in AMD. In the process of ocular neovascularization, integrin receptor α_V_β_3_ was over expressed on the surface of RPE [27,28,29], and Arg-Gly-Asp (RGD) moiety containing peptide can specifically bind to integrin receptor [22,30,31]. The cyclic RGD (cRGD) peptide has a particularly high affinity with α_V_β_3_ [32]. As a new drug delivery carrier, PLGA nanoparticles have the advantages of high biocompatibility and suitable size for cell uptake [33]. Therefore, nano-drug delivery system for co-encapsulate dexamethasone and bevacizumab modified with cRGD peptide is a promising treatment for AMD.

In the present work, we prepared the dexamethasone-loading cRGD-PEG-PLGA/PLGA/PEI nanoparticles (cRGD-DPPNs); firstly, cRGD-PEG-PLGA was added as the target to the RPE and the branched polyethylenimine (PEI) was used to regulate the potential. The adsorption of negative bevacizumab and the positive cRGD-DPPNs was to form bevacizumab-adsorbing cRGD-DPPNs (aBev/cRGD-DPPNs). Then the nanoparticles were evaluated by a series of physicochemical characterizations, in vitro stability and release behavior assay. The anti-angiogenic ability of nanoparticles to human umbilical vein epithelial cells (HUVECs) and their uptake by adult retinal pigment epithelial cells (ARPE-19) were studied. Finally, the therapeutic effect of nanoparticles on CNV was investigated by establishing CNV chinchilla rabbit model.

## 2. Results and Discussion

### 2.1. Physicochemical Characterization of the aBev/cRGD-DPPNs

**Figure 1:**
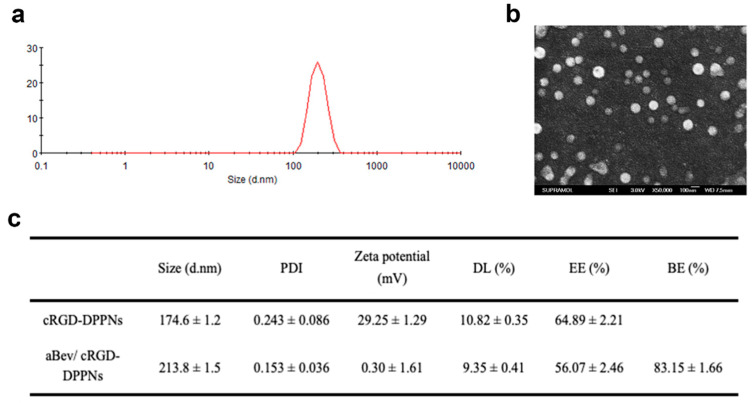
Physicochemical characterization of the bevacizumab-adsorbing and dexamethasone-loading cRGD-PEG-PLGA/PLGA/PEI nanoparticles (aBev/cRGD-DPPNs). (**a**) Dynamic light scattering (DLS) of the aBev/cRGD-DPPNs; (**b**) scanning electron microscope (SEM) of the aBev/cRGD-DPPNs; (**c**) characteristics of the aBev/cRGD-DPPNs and the dexamethasone-loading cRGD-PEG-PLGA/PLGA/PEI nanoparticles (cRGD-DPPNs).

PLGA was used as the carrier material of nanoparticles, and the branched PEI was used to regulate the potential of nanoparticles, meanwhile, cRGD peptide was regarded as the target for preparing cRGD-DPPNs loaded with dexamethasone. The aBev/cRGD-DPPNs were prepared by electrostatic adsorption of the negative bevacizumab to the surface of the positive cRGD-DPPNs. Dynamic light scattering (DLS) and scanning electron microscope (SEM) images of the aBev/cRGD-DPPNs were shown in Figure 1a,b, respectively. The SEM results showed that the nanoparticles were smooth, spherical, and uniform in size. The optimal aBev/cRGD-DPPNs showed 213.8 ± 1.5 nm in particle size, 0.153 ± 0.036 in polydispersity index (PDI), and 0.30 ± 1.61 mV in potential. Drug loading (DL) (%), encapsulation efficiency (EE) (%), and binding efficiency (BE) (%) of the aBev/cRGD-DPPNs were 9.35 ± 0.41%, 56.07 ± 2.46%, and 83.15 ± 1.66% (Figure 1c), respectively. The particles with a diameter of about 200 nm exhibited significantly enhanced ocular posterior segment tissues accumulation than with the other size [34].

### 2.2. Stability of the aBev/cRGD-DPPNs

**Figure 2:**
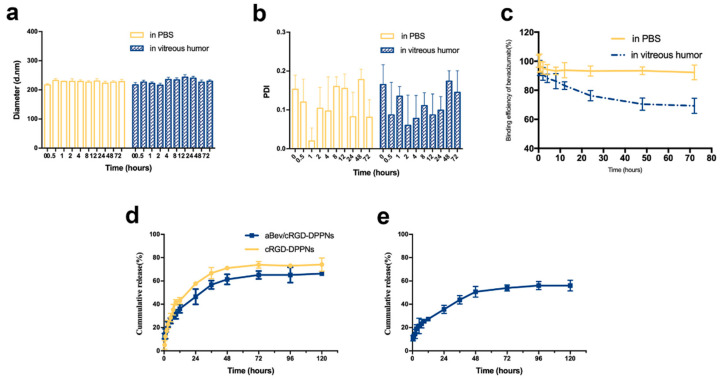
Stability and release behavior of the aBev/cRGD-DPPNs. (**a**) Stability of particle size change; (**b**) stability of polydispersity index (PDI) change; (**c**) stability of binding efficiency (BE) change; (**d**) in vitro release of dexamethasone from the aBev/cRGD-DPPNs and the cRGD-DPPNs; (**e**) in vitro release of bevacizumab from the aBev/cRGD-DPPNs. Data are expressed as mean ± SD, *n* = 3.

The aBev/cRGD-DPPNs were resuspended in PBS of pH 7.4 and fresh rabbit eye vitreous to investigate the stability at 37 °C. The results of particle size, PDI, and BE change of the aBev/cRGD-DPPNs were shown in Figure 2a–c. The results showed that the aBev/cRGD-DPPNs had good dispersion stability in 72 h under PBS condition. However, under the condition of rabbit vitreous humor, the particle size of the aBev/cRGD-DPPNs increased within 4–48 h and decreased over time, which was due to the possibility that proteins from the vitreous might adsorb to the nanoparticles, resulting in the increase of the size. As time going on, the interaction between nanoparticles and vitreous proteins may become stronger, which may lead to more compact structures and smaller size. PDI value fluctuated slightly but never exceeded 0.3. The change rate of BE decreased gradually within 72 h under the vitreous condition. A small amount of bevacizumab slicing (about 30%) and the various proteins in vitreous humor will affect the particle size. The shedding of bevacizumab indicates that it will release from the aBev/cRGD-DPPNs into the vitreous body in vivo and exert its efficacy.

### 2.3. Sequentially Release of Dexamethasone and Bevacizumab In Vitro

The in vitro release profiles of dexamethasone and bevacizumab from the aBev/cRGD-DPPNs and the cRGD-DPPNs in release medium were investigated. The release result of dexamethasone was shown in Figure 2d. At 12 h, the burst release of dexamethasone from the aBev/cRGD-DPPNs and the cRGD-DPPNs was 36.16 ± 3.52% and 43.05 ± 2.72%, respectively. The cumulative release from the aBev/cRGD-DPPNs and the cRGD-DPPNs was 66.32 ± 1.38% and 74.01 ± 5.72% at 120 h, respectively.

The result of bevacizumab release was shown in Figure 2e. At 12 h, the burst release of bevacizumab from the aBev/cRGD-DPPNs was 27.26 ± 1.54%. At 12 h, the burst release of bevacizumab from the aBev/cRGD-DPPNs was 56.20 ± 4.59%. The subsequent release of dexamethasone and bevacizumab from the aBev/cRGD-DPPNs could expose drugs to the CNV area with a long-term effective drug concentration.

### 2.4. Cellular Uptake of the aBev/cRGD-DPPNs

**Figure 3:**
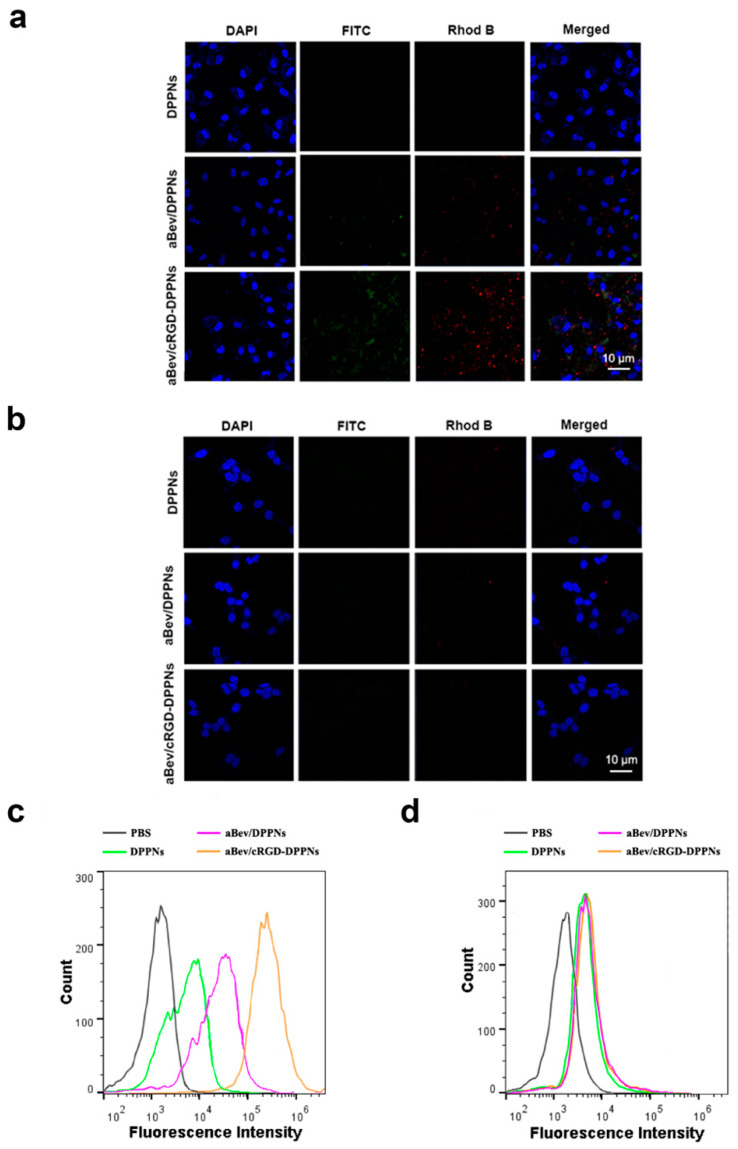
Cellular uptake of the aBev/cRGD-DPPNs. (**a**) Uptake of nanoparticles in ARPE-19 cells by Confocal laser scanning microscopy (CLSM); (**b**) uptake of nanoparticles in 293T cells by CLSM; (**c**) uptake of nanoparticles in ARPE-19 cells by Flow cytometry (FCM); (**d**) uptake of nanoparticles in 293T cells by FCM.

Confocal laser scanning microscopy (CLSM) was used to observe the drug distribution in ARPE-19 cells and 293T cells after internalization of nanoparticles. Compared with the overexpression of integrin α_v_β_3_ in ARPE-19 cells, the surface of 293T cells had an extremely low expression of integrin α_v_β_3_ [35]. We used double fluorescent labeled nanoparticles to investigate the degree of cell internalization of nanoparticles. The results are shown in Figure 3a,b. The blue fluorescence represents DAPI-labeled cell nucleus. The red fluorescence was the core of the DPPNs, the aBev/DPPNs or the aBev/cRGD-DPPNs labeled by hydrophobic Rhod B. FITC-labeled bevacizumab shell was with green fluorescence. In ARPE-19 cells, it can be seen from the merged image that the nucleus of the aBev/cRGD-DPPNs group was surrounded by red and green fluorescence, which proves that the aBev/cRGD-DPPNs were internalized into the cell nearly at the same time, and bevacizumab did not dissociate during the process of cell internalization. However, the fluorescence intensity of the DPPNs and the aBev/DPPNs in ARPE-19 cells was very weak, which indicates that the aBev/cRGD-DPPNs has higher cellular internalization efficiency, and the aBev/cRGD-DPPNs can target ARPE-19 cells more effectively. However, no obvious red and green fluorescence was observed in 293T cells, indicating that the aBev/cRGD-DPPNs have a targeting selectivity for the ARPE-19 cells over expressing integrin α_v_β_3_.

Flow cytometry (FCM) was used to investigate the internalization efficiency of nanoparticles in ARPE-19 cells and 293T cells. Since the two fluorescence distributions measured by CLSM were similar, only Rhod B was used to mark the nanoparticles in this experiment. As seen in Figure 3c, the curve of the aBev/cRGD-DPPNs shifted significantly to the right, and the degree of cell internalization was positively correlated with the fluorescence intensity of Rhod B, indicating that the aBev/cRGD-DPPNs were absorbed by ARPE-19 cells more than the DPPNs even the aBev/DPPNs. However, there was no significant difference in Rhod B fluorescence intensity of the DPPNs, the aBev/DPPNs and the aBev/cRGD-DPPNs in 293T cells (Figure 3d), which showed that the enhanced cell internalization efficiency of the aBev/cRGD-DPPNs in ARPE-19 cells was attributed to the targeted delivery of nanoparticles. The enhanced association of aBev/cRGD-DPPNs compared to unmodified aBev/DPPNs was contributed by cRGD. This result is consistent with those of CLSM.

### 2.5. Apoptosis, Wound Healing, Transwell Invasion, and Tube Formation Assay of HUVECs

**Figure 4:**
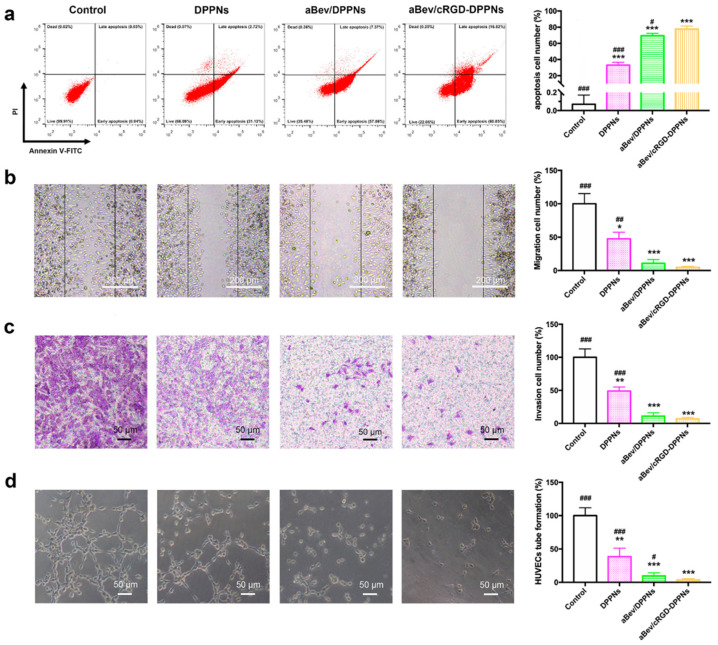
The aBev/cRGD-DPPNs induced apoptosis and inhibited migration, invasion, and tube formation of HUVECs. (**a**) The aBev/cRGD-DPPNs induced apoptosis of HUVECs; (**b**) the aBev/cRGD-DPPNs inhibited HUVECs migration in wound healing assay; (**c**) the aBev/cRGD-DPPNs inhibited HUVECs invasion in Transwell assay; (**d**) the aBev/cRGD-DPPNs inhibited tube formation of HUVECs. Data are expressed as mean ± SD, *n* = 6. * *p* < 0.05, ** *p* < 0.01, *** *p* < 0.001 vs. the control; # *p* < 0.05, ## *p* < 0.01, ### *p* < 0.001 vs. the aBev/cRGD-DPPNs.

Endothelial cell apoptosis plays a critical role in the angiogenic process. FITC annexin V/PI apoptosis detection kit was used to evaluate the induction of apoptosis by nanoparticles. As shown in Figure 4a, there was almost no apoptosis in the control group. The apoptosis percent of the DPPNs, the aBev/DPPNs, and the aBev/cRGD-DPPNs was 33.04 ± 3.53%, 69.53 ± 2.87%, and 77.63 ± 3.82%, respectively. The results showed that the aBev/cRGD-DPPNs can significantly induce apoptosis of HUVEC cells (*p* < 0.001).

Endothelial cell migration and invasion are crucial steps in angiogenesis. The inhibition of the nanoparticles on endothelial cell migration and endothelial cell invasion was evaluated by wound healing assay and Transwell assay. We found that all the DPPNs, the aBev/DPPNs, and the aBev/cRGD-DPPNs inhibited migration of HUVECs (Figure 4b), and the aBev/cRGD-DPPNs showed a smaller migration effect than the DPPNs and the aBev/DPPNs (47.68 ± 9.88% vs. 4.73 ± 1.57%, *p* < 0.01; 10.95 ± 5.62%). In Figure 4c, the aBev/cRGD-DPPNs showed a smaller invasion effect than the DPPNs and the aBev/DPPNs (49.17 ± 5.98% vs. 6.81 ± 2.36%, *p* < 0.001; 11.23 ± 4.80%).

During the late stage of angiogenesis, after the endothelial cells have finished migrating and invading, the endothelial cells will further arrange into tube-like structures. We further evaluated the inhibitory effect of nanoparticles on the ability of tube formation on matrix gel. The results in Figure 4d showed the aBev/cRGD-DPPNs induced a stronger HUVECs tube formation inhibitory effect than the DPPNs (*p* < 0.001) and the aBev/DPPNs (*p* < 0.05). These results illustrated that the aBev/cRGD-DPPNs had significant anti-angiogenic effects on HUVECs.

### 2.6. In Vivo CNV Inhibition Study

The CNV growth inhibition study was studied by fluorescein fundus angiography (FFA). FFA was conducted 4 weeks after administration. The contrast agent of 10% fluorescein sodium (0.2 mL) and 0.9% saline (1 mL) were injected through the peripheral vein of the ear. FFA photo was recorded with high performance digital imaging system (Heidelberg HRA-II, Germany).

**Figure 5:**
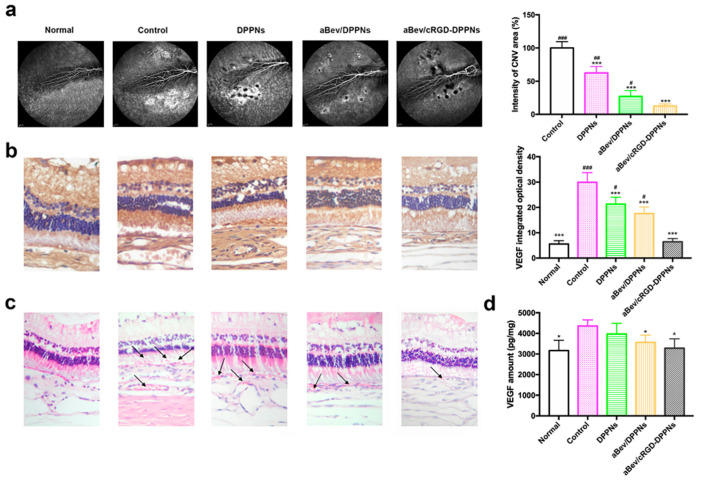
The aBev/cRGD-DPPNs inhibited choroidal neovascularization (CNV) development in vivo. (**a**) The aBev/cRGD-DPPNs decreased the area of CNV leakage by fluorescein fundus angiography (FFA); (**b**) immunohistochemical results of the aBev/cRGD-DPPNs treated CNV rabbits; (**c**) histopathology analysis of the aBev/cRGD-DPPNs treated CNV rabbits; (**d**) VEGF amount in the aBev/cRGD-DPPNs treated CNV rabbits. Data are expressed as mean ± SD, *n* = 3. * *p* < 0.05, *** *p* < 0.001 vs. the control; # *p* < 0.05, ## *p* < 0.01, ### *p* < 0.001 the aBev/cRGD-DPPNs.

FFA was performed to examined CNV development. Four weeks after intravitreal injection of the drugs, the laser lesions of fluorescent leakage were evaluated by FFA (Figure 5a). Large and diffuse areas of leakage were observed in the eyes of thenon-treated CNV group. The intensity of fluorescent leakage intensity in the DPPNs, the aBev/DPPNs, and the aBev/cRGD-DPPNs group was 62.53 ± 9.66%, 27.17 ± 8.82%, and 12.58 ± 3.12%, respectively. The inhibitory effects of the aBev/cRGD-DPPNs on the area of CNV leakage were statistically significant (*p* < 0.001).

Immunohistochemistry results of VEGF protein expressed in the retina-choroid appeared brownish-yellow staining in the slices, and the darker of brownish. IOD values of the positive staining area were positively correlated with VEGF expression, as shown in Figure 5b. The IOD value of the normal group, the control group, the DPPNs, the aBev/DPPNs, and the aBev/cRGD-DPPNs group was 5.58 ± 1.30, 29.91 ± 3.79, 21.31 ± 2.72, 17.59 ± 2.58, and 6.46 ± 1.24, respectively. The expression of VEGF in the control group was significantly higher than that in the normal group (*p* < 0.001). The aBev/cRGD-DPPNs showed the most significant inhibitory effect on VEGF (*p* < 0.001).

HE staining results of the retina-choroid tissue are shown in Figure 5c. The structure of normal tissue was complete and clear. In the CNV control group, there were obvious nuclear condensation in the inner and outer nuclear layers, cell atrophy in the photoreceptor layer, scar formation in the RPE cell layer toward the retina, and obvious growth of CNV (black arrows). After administration of the DPPNs, the aBev/DPPNs or the aBev/cRGD-DPPNs, the typical CNV structure was reduced, and the CNV lesions structure showed a significantly decreased in the aBev/cRGD-DPPNs group.

Previous studies have shown that VEGF is mainly expressed during the embryonic stage, plays an important role in the formation and development of blood vessels. Furthermore, VEGF can be expressed in the process of angiogenesis. Therefore, the expression level of VEGF protein in RPE-choroidal complex was quantitatively analyzed by ELISA, as shown in Figure 5d. The content of VEGF in RPE choroidal complex of CNV rabbit model can be effectively reduced by intravitreal administration of the aBev/cRGD-DPPNs (*p* < 0.05).

## 3. Materials and Methods

### 3.1. Materials

Dexamethasone and FITC were purchased from Yuanye Biotechnology (Shanghai, China). Bevacizumab (molecular weight (MW) 149 kDa) was donated by Luye Pharmaceutic (Yantai, China). PLGA 503H was purchased from Evonic Industries (Birmingham, AL, Germany). cRGD-PEG-PLGA was purchased from Xi’an Ruixi biotechnology (Xi’an, China). Micro bicinchoninic acid (BCA) protein assay kits were purchased from Thermo Scientific (Waltham, MA, USA). PEI (MW 25 kDa, branched) and polyvinyl alcohol (PVA) (MW 13–23 kDa) were purchased from Sigma-Aldrich (St Louis, MO, USA). Rhod B was purchased from Sinopharm chemical reagents (Beijing, China). Acetonitrile was purchased from Thermo Fisher Scientific (Waltham, MA, USA). Rabbit VEGF enzyme-linked immunosorbent assay (ELISA) kits were obtained from Elabscience Biotechnology (Wuhan, China). All other chemicals used were of analytical grade and used without further purification.

### 3.2. Preparation of the aBev/cRGD-DPPNs

The cRGD-DPPNs containing PLGA and cRGD-PEG-PLGA with a proportion of 9:1 (*w*/*w*) were prepared by an emulsion solvent volatilization method. Briefly, PLGA (22.5 mg) together with cRGD-PEG-PLGA (2.5 mg) were dissolved in 1.5 mL chloroform and mixed with dexamethasone (6 mg) dissolved in 1 mL acetone. The above mixed solution was added to 1% PVA (*w*/*v*) aqueous solution containing PEI with positive charge dropwise and was subject to phacoemulsification. The cRGD-DPPNs were formed and dried with a magnetic stirrer at 25 °C over night to remove the residual solvent completely. Meanwhile, the preparation of DPPNs was the same as that of cRGD-DPPNs except that the cRGD-PEG-PLGA was replaced by an equal amount of PLGA.

The aBev/cRGD-DPPNs were prepared by the adsorption method based on the method reported before [36]. A certain mass of bevacizumab and the cRGD-DPPNs were co-dispersed in PBS (pH 8.0) for 2 h at 25 °C. Ultrafiltration tubes (MWCO 300 kDa) were used to remove unconnected bevacizumab and salt ions from the solution. Freeze-dried after −20 °C storage in the dark reserve. The final products were lyophilized.

The bevacizumab-adsorbing dexamethasone-loading PLGA/PEI nanoparticles (aBev/DPPNs) were fabricated for comparison.

### 3.3. Physicochemical Characterization of the aBev/cRGD-DPPNs

The size, distribution, and potential of the nanoparticles were measured by ZS90 dynamic light scattering (DLS) (Malvern, UK).

Then the morphological characteristics of the aBev/cRGD-DPPNs were observed by scanning electron microscope (SEM) (JXA-840, JEOL, Tokyo, Japan) sample platform. The aBev/cRGD-DPPNs were diluted with deionized water to 1 mg/mL, respectively. The nanoparticles suspension was dropped on the clean silicon wafer, and the surface liquid was dried overnight at room temperature. The silicon wafer was fixed and sprayed with gold in a vacuum for 20 s. The test voltage was set to 3 kV.

The dexamethasone encapsulation efficiency (EE) into the aBev/cRGD-DPPNs were detected using HPLC (Waters, Milford, MA, USA) at 240 nm. The mobile phase composed of acetonitrile and water at a ratio of 60:40 (*v*/*v*) was loaded onto an Agilent XDB-C18 column (Agilent Technologies, Lexington, MA, USA, 4.6 mm × 250 mm, 5 μm). Briefly, the content of dexamethasone was evaluated via dissolving the aBev/cRGD-DPPNs in 1 mL of acetonitrile and subsequently diluted to 5 mL with the mobile phase. The drug loading (DL) was calculated as the ratio of the amount of dexamethasone encapsulated in the nanoparticles to the total amount of the nanoparticles. The EE was calculated as the ratio of the actual dexamethasone loading to the theoretical dexamethasone loading. The bevacizumab binding efficiency (BE) in the aBev/cRGD-DPPNs was examined via collecting the solution removed from ultrafiltration tubes centrifugation and quantitively analyzed by a Micro BCA protein assay. The BE was calculated as the ratio of the amount of total bevacizumab subtract free bevacizumab to the total amount of the nanoparticles.

### 3.4. Stability of the aBev/cRGD-DPPNs

To investigate the stability of the aBev/cRGD-DPPNs under in vitro physiological conditions, the aBev/cRGD-DPPNs were suspended in PBS of pH 7.4 and fresh rabbit eye vitreous, respectively, and then stored at 37 °C. The particle size, PDI, and BE were measured at predetermined time with the methods mentioned above.

### 3.5. Sequentially Release of Dexamethasone and Bevacizumab In Vitro

The nanoparticles were suspended in 1 mL release medium of PBS containing 0.5% Tween-80 of pH 7.4. Then the dialysis bag (MWCO 8 kDa) containing mixed suspension was put into the centrifuge tube containing 50 mL same release medium in a shaker at 37 °C with 110 rpm. At pre-specified time intervals, 2 mL of releasing medium was taken out, and the equal volume fresh releasing medium was added. The dexamethasone amount was determined by HPLC and the bevacizumab amount was determined by the Micro BCA protein assay.

### 3.6. Cellular Uptake of the aBev/cRGD-DPPNs

The cellular uptake of nanoparticles was performed using fluorescently labeled nanoparticles and measured by confocal laser scanning microscopy (CLSM) and flow cytometry (FCM) analysis. Dexamethasone was replaced by fluorescent dye Rhod B, and Bev was labeled with FITC. Briefly, Bev and FITC had the proportion of 1:7 (*w*/*w*). The FITC solution was added to the Bev solution dropwise and the reaction was stopped by 5 M NH_4_Cl. The reaction solution was dialyzed overnight to remove unreacted FITC. The α_V_β_3_-positive ARPE-19 cells were incubated at 37 °C in a 5% CO_2_ in RPMI 1640 (Hyclone, Logan, UT, USA) with 10% FBS (Procell, Wuhan, China) and 1% penicillin/streptomycin (Gibco, Grand Island, NE, USA). The α_V_β_3_-negative Human embryonic kidney cells (293T) were incubated at 37 °C in a 5% CO_2_ in DMEM (Gibco) with 10% FBS and 1% penicillin/streptomycin.

ARPE-19 cells and 293T cells in the log growth stage were seeded into 12-well plates containing climbing tablets and cultured for 24 h making the cells grow to 75%. The original cell culture medium was discarded, and the fluorescently-labeled the DPPNs, the aBev/DPPNs, the aBev/cRGD-DPPNs were added to continue the dark culture for 2 h. Then, the cells were rinsed gently 3 times with PBS, and fixed with Verhoeff’s solution for 15 min. Then, the cells were treated with Triton X-100 for 5 min to increase cell permeability, stained cellular nuclei with DAPI dye solution, and imaged under CLSM (LSM710, Carl Zeiss, Berlin, Germany).

To quantify the uptake of nanoparticles by ARPE-19 cells and 293T cells, the cells in the log growth stage were seeded into 6-well plates and placed in a 5% CO_2_ 37 °C incubator for 24 h. The original cell culture medium was discarded and Rho B-labeled the DPPNs, the aBev/DPPNs or the aBev/cRGD-DPPNs were added. The cells were incubated for an additional 4 h and rinsed gently with PBS 3 times, 200 μL of trypsin cells were added to each well, and the cells were re-suspended in the pre-cooled PBS, and the collected cells were examined by FCM (Beckman Coulter, Brea, CA, USA) to determine the cellular uptake efficiency of the nanoparticles.

### 3.7. Apoptosis, Wound Healing, Transwell Invasion, and Tube Formation Assay of HUVECs

HUVECs were incubated at 37 °C in a 5% CO_2_ in Medium 199 (Gibco) supplemented with 10% FBS and 1% penicillin/streptomycin. The DPPNs, the aBev/DPPNs and the aBev/cRGD-DPPNs were suspended in serum-free culture medium for cell cultures.

HUVECs were collected and cultured in 6-well plates. After growing to 80%, the starved cells were treated with drugs (the DPPNs, the aBev/DPPNs or the aBev/cRGD-DPPNs). After 24 h of treatment, 50,000~100,000 cells were taken, the supernatant was centrifuged and Annexin V-FITC and PI staining solution were added and gently mixed. Following incubation for 15 min, the cells were washed and analyzed by FCM.

The wound healing assay was performed as described previously [37]. HUVECs were cultured into 12-well plates. After the cells grew full, the monolayer HUVECs were scratched with 20 μL straws tips. Serum-free medium containing the DPPNs, the aBev/DPPNs or the aBev/cRGD-DPPNs was added respectively, and the pure medium was used as control. The migrated cells were quantified (CKX41, Olympus, Tokyo, Japan) by artificial counting after 24 h. The inhibition rate was calculated based on 100% of HUVECs treated with the medium.

The inhibition of the nanoparticles on cell invasion was evaluated by Transwell invasion assay. The Transwell chambers (8 μm microporous) were placed in 24-well plates, and the top chamber was added with 50 μL matrix gel on the ice, and kept at 37 °C for 10 min. In the top chamber, 200 μL of 4 × 10^4^ HUVECs in the pure medium containing the DPPNs, the aBev/DPPNs, or the aBev/cRGD-DPPNs were added. The plate wells with the chambers were filled with medium containing VEGF165 medium. HUVECs were allowed to migrate for 24 h. The cells were fixed with 100% methanol, washed with PBS for three times, then stained with 0.05% crystal violet. Then the invasive cells were counted and quantified, and the cells treated with simple medium were set as 100%.

The tube formation assay of capillary-like structures inhibition was performed as described previously [36,38]. The 300 μL matrix gel (8.6 mg/mL) was added to the 24-well plates on ice and kept at 37 °C for 30 min to polymerize into the membrane. Then HUVECs with the DPPNs, the aBev/DPPNs, or the aBev/cRGD-DPPNs in the pure medium were sown to the matrix gel. After 6 h, images were taken under 20 × inverted microscope and analyzed by Image J software.

### 3.8. The Establishment of Rabbits CNV Model

Male chinchilla rabbits weighting 2.5~3.0 kg used in this experiment were approved by the Ethical Committee for Care and Use of Laboratory Animals at Jilin University (Ethics approval number: SY201904012; Ethics approval date: 30 June 2019). All rabbits were cared for and treated in accordance with legal and institutional guidelines. The chinchilla rabbits with no abnormalities in the anterior segment and the fundus of the eyes were randomized into five groups: (1) Normal group: normal rabbits injected with 100 μL 0.9% saline; (2) Control group: CNV rabbits injected with 100 μL 0.9% saline; (3) DPPNs group: CNV rabbits injected with 100 μL DPPNs containing 0.1 mg dexamethasone; (4) aBev/DPPNs group: CNV rabbits injected with 100 μL aBev/DPPNs containing 0.1 mg dexamethasone; (5) aBev/cRGD-DPPNs group: CNV rabbits injected with 100 μL aBev/cRGD-DPPNs containing 0.1 mg dexamethasone. The drug suspension was intravitreally injected to the right eyes with 27-needle 4 weeks after laser photocoagulation.

CNV model was induced by laser in chinchilla rabbits. Briefly, the healthy chinchilla rabbits were anesthetized with 10% chloral hydrate. Alcaine^®^ and Mydriacyl^®^ were following eye drops to the right eyes for conjunctival surface anesthesia and mydriasis. Then, the laser photocoagulation was carried out with an argon green laser with a wavelength of 532 nm (power 0.7 W, exposure time 0.1 s, 20 spots with diameter 50 μm, with the degree of bubble generation after photocoagulation, the bubble generation indicated that Bruce membrane rupture) (532 nm; Iris Radiation Systems, Povo, Italy).

### 3.9. In Vivo CNV Growth Inhibition Study

The CNV growth inhibition study was studied by fluorescein fundus angiography (FFA). FFA was conducted 4 weeks after administration. The methods of anesthesia and mydriasis were the same as described in 3.8. The contrast agent of 10% fluorescein sodium (0.2 mL) and 0.9% saline (1 mL) was injected through the peripheral vein of the ear. FFA photo was recorded with high performance digital imaging system (Heidelberg HRA-II, Germany).

### 3.10. In Vivo Immunohistochemistry and Histopathological Evaluation in RPE-Choroid

Three rabbits in each group were killed by vein embolization at the ear edge after 4 weeks of vitreous administration. The right eyeball was removed and fixed in Verhoeff’s solution for 24 h to enable a histological evaluation. The embedded eyeballs were sectioned continuously parallel to the sagittal axis of the optic nerve with a thickness of 5 μm. Slices of RPE-choroid were roasted in a 60 °C incubator for at least 1 h. After dewaxing and hydration, the slices were filled with EDTA buffer solution and placed in a microwave oven for antigen repair. Subsequently, the slices were incubated with goat serum at 37 °C for 10 min, followed by a VEGF antibody at 37 °C for 2 h and rinsed 3 times with TBS-T. The slices were subsequently blocked by goat serum at 37 °C for 10 min, followed by a biotinylated secondary antibody (1/300, goat) at 37 °C for 30 min, and washed 3 times with TBS-T. The slices were subsequently sealed with Tween 20 and incubated with HRP-SA at 37 °C for 30 min. Finally, after TBS-T washing 3 times and TBS washing 5 times, the slices were stained with 3,3′-diaminobenzidine (DAB) and counterstained with hematoxylin. DAB tan color rendering was positive. The sections were imaged using a light microscope (Nikon Instruments Inc., Tokyo, Japan). The integrated optical density (IOD) of the positive staining area under 400× optical microscope was calculated by using Motic Images Advanced 3.2 software.

On the other hand, slices of RPE-choroid were stained with H&E prior to histopathological observation of the retinas. A light microscope (Nikon Instruments Inc., Tokyo, Japan) at 200× magnification was used to examine the slices.

### 3.11. In Vivo Downregulation of VEGF Protein

VEGF protein amount in RPE-choroid was quantified using a Rabbit VEGF ELISA kit in accordance with manufacturer’s instructions. ODs were measured at 450 nm (Multiskan Spectrum, Thermo, Waltham, MA, USA).

### 3.12. Statistical Analysis

All data are expressed as mean ± standard deviations (SD). A one-way analysis of variance followed by the t-test was applied to all statistical comparisons. A *p*-value < 0.05 was considered to indicate a significant difference.

## 4. Conclusions

The aBev/cRGD-DPPNs in this study showed size uniform and good biocompatible. In vitro uptake experiments showed that cRGD peptide could effectively promote the uptake of nanoparticles by ARPE-19. It can effectively inhibit the inhibition of angiogenesis in vivo and in vitro and has a good therapeutic effect on rabbit CNV in vivo.

In conclusion, the drug co-delivery nano carrier modified with cRGD peptide in this study can effectively deliver drugs to the site of AMD and exert effects, and the combination of dexamethasone and bevacizumab can better inhibit CNV. This study presents an ideal carrier, a new idea of combination medication, and targeted therapy for the treatment of AMD.
